# Paracrine activity of adipose derived stem cells on limbal epithelial stem cells

**DOI:** 10.1038/s41598-021-99435-1

**Published:** 2021-10-07

**Authors:** Bartosz Sikora, Aleksandra Skubis-Sikora, Agnieszka Prusek, Joanna Gola

**Affiliations:** 1 Department of Cytophysiology, Chair of Histology and Embryology, Faculty of Medical Sciences in Katowice, Medical University of Silesia in Katowice, ul. Medyków 18, C2/103, 40-752 Katowice, Poland; 2 Department of Molecular Biology, Faculty of Pharmaceutical Sciences in Sosnowiec, Medical University of Silesia in Katowice, Katowice, Poland

**Keywords:** Stem-cell research, Stem cells, Regeneration

## Abstract

Limbal stem cells deficiency (LSCD) is an eye disease caused by the loss of stem cells in the corneal limbus as a succession of an injury due physical, biological, or chemical agents. Current therapies of LSCD are focused on the transplantation of donor corneas or tissue equivalents produced from autologous limbal stem cells. Every year there are waiting millions of patients for the cornea transplantation all over the world and the list is growing due to the relatively low number of cornea donors. On the other hand, the transplantation of tissue or cells into the recipient’s body is associated with the higher risk of possible side effects. The possibility of the application of an indirect treatment using the properties of the paracrine activity of stem cells, would be beneficial for the patients with transplant failures. This study was to evaluate the paracrine effect of mesenchymal stem cells derived from adipose tissue (ADSC) on the viability of limbal epithelial stem cells (LESC). The paracrine effect was assessed by treating LESC with conditioned medium collected from ADSC culture. Cell viability, cytotoxicity, apoptosis and proliferation were evaluated using in vitro assays in standard conditions and induced inflammation. After the exposure to the examined conditions, the expression of genes related to pro- and anti- inflammatory factors was evaluated and compared to the secretion of selected cytokines by ELISA test. Moreover, the changes in LESC phenotype were assessed using of phenotype microarrays. Our findings suggest that paracrine activity of ADSC on LESC promotes its proliferation and has a potential role in mitigation of the adverse impact of inflammation induced by lipopolysaccharide.

## Introduction

Cornea is front, transparent part of eye responsible for transmission and refraction of light. It possesses high self-regenerative ability due to the presence of limbal epithelial stem cells (LESC) what makes the cornea easy to be transplanted. LESC are localized in corneal limbus which is a thin, transparent cell zone in the junction between cornea, sclera and conjunctiva^1^. Limbal Stem Cell Deficiency (LSCD) may occur due to chemical or thermal burns and physical destruction. LSCD is also a congenital disease like aniridia-associated keratopathy (AAK), corneal leukoma or Fuchs’ dystrophy^2–4^.

Cornea is one of the most frequently transplanted organs. Unfortunately, despite the possibility of transplantation, insufficient number of donors in comparison to number of recipients is still a huge problem of transplant medicine. Statistical report of Eye Bank Association of America (EBAA) showed that 85,441 corneal transplantations were done in year 2018^5^. Reported cases of corneal transplantations in 2019 reached 85,601. In 2020 due the worldwide pandemic of COVID-19 reported procedures dramatically decreased to the number of 66,278 surgeries. The pandemic has influenced on performing many surgical procedures and the implemented sanitary restrictions in most countries all over the world had slower healthcare system what appeared in lower number of all surgical procedures. Instead of the successfully ended cornea transplantation reported by EBAA, every year there is a huge number of waiting patients for the corneal transplantation all over the world. In 2016 it was reported about 12.7 million of waiting patients^6^.

Treatment of the corneal disorders may embrace partial or whole cornea transplantation. Unfortunately, the number of patients waiting for the graft indicates that there are not enough cornea donors, thus newer and newer medicinal products for corneal reconstruction are being introduced. When other treatment fails, standard surgical procedure of damaged cornea replacement with a donor tissue is being displaced by artificial cornea transplantation^7–10^. As well the use of xenogeneic corneas is being considered in the absence of cornea donors^11–13^. However, each medicinal product of such matter is produced with active stem cells usually harvested from small biopsy taken from the healthy limbal region of patients’ eye. Such a biopsy could potentially broaden stem cells deficiency and cause extensive damage to the corneal epithelium. On the other hand, not all the corneal injures require immediate replacement. New strategies may focus on improving residual limbal stem cells viability and supporting them with factors or drugs aimed to activate their proliferation and thus tissue reconstruction. Therefore, new strategies on corneal treatment, based on supporting the residual stem cells should be developed. Regarding these concerns we assume that new strategy of corneal treatment which will be based on supporting residual healthy stem cells niche in patients’ eye will be the future therapy of limbal stem cells deficiency.

Mesenchymal stem cells (MSCs) are somatic cells with wide regenerative properties. Research focus on using them e.g. in bone and cartilage failures regeneration or cardiovascular disease therapies. One of the common sources of MSCs is adipose tissue. Adipose tissue is a rich source of mesenchymal stem cells. Its collection is an easy procedure and the probable amount of stem cells which can be gathered during the isolation procedure is high. Considering other sources of mesenchymal stem cells, liposuction is burdened with low risk of complications in opposite to the bone marrow biopsy. Mesenchymal stem cells, besides its plasticity, exhibit immunomodulatory abilities by secreting numerous cytokines^14,15^. ADSC affects neighboring cells also via secretion of growth factors^16,17^ and extracellular vesicles^18–20^. Secretome of ADSC promotes proliferation and reduces inflammation^21,22^. MSCs secrete anti-scarring factors as KGF, SDF-1, MIP-1a, MIP-1b, anti-apoptotic agents as STC-1, SFRP2, TGF-β1, VEGF and HGF, molecules associated to angiogenesis like VEGF, TGF-β1 and mitogenic factors as TGF-α, TGF-β, HGF, IGF-1, FGF-2 or EGF^15,23^. Research indicated that MSCs secretome act positively on corneal epithelium^24^. It suggests that ADSC can be used clinically of example in the treatment of LSCD.

Inflammation is accompanying most corneal disorders. Our experiment evaluates the paracrine activity of ADSC on LESC in the in vitro model of inflammation. This kind of evaluation can give quick answer if ADSC’s secreted factors act positively on cells under the induced inflammation, what can be useful in studies on potential new therapeutic agents in most corneal disorders. In this report, the effect of ADSC’s secreted factors on the proliferation, metabolic activity, and transcriptome of limbal stem cells was analyzed under the condition of inflammation induced by bacterial lipopolysaccharide (LPS).

## Material and methods

### Experiment

Paracrine activity of ADSC was assessed in an in vitro model of LESC dysfunction. Experiment was conducted in two aspects. First, the direct influence of conditioned medium (CM) from ADSC on LESC activity was assessed. The examined cells were compared to the control cells cultured in standard medium (SM). Then, LESC were pretreated with LPS to induce inflammation and again cultured in CM to assess its influence under the condition of induced inflammation (CM_LPS group). The control cells were cultured in SM after analogous pretreatment with LPS (SM_LPS group).

### Cell culture conditions

ADSC (PT-5006) were obtained from Lonza company (Switzerland) and LESC were provided by courtesy of Department of Microbial Biotechnology and Cell Biology, Faculty of Science, University of Debrecen (Hungary) where they were isolated and identified^25^. ADSC and LESC were cultured in a Dulbecco's Modified Eagle Medium (DMEM, Lonza, Switzerland) with 10% of fetal bovine serum (FBS, EuroClone, Italy) and 1% of antibiotics: amphotericin B with penicillin–streptomycin (Lonza, Switzerland) at 37 °C in a 5% CO_2_ incubator (Direct Heat CO_2_; Thermo Fisher Scientific, USA). The medium was changed every 48 h. Identification of ADSC was made with real time RTqPCR analysis of genes: *CD73*, *CD90* and *CD105* and flow cytometry with Human Mesenchymal Stem Cell Marker Verification Multi-Color Flow Cytometry Kit for the analysis of surface proteins: CD73, CD90 and CD105 (R&D Systems, USA). Cell fluorescence was measured with FACS Aria 2 (Becton Dickinson, USA) and the data was analyzed using software (Becton Dickinson, USA) (not shown).

### Conditioned medium preparation

CM was collected from 24 h culture of ADSC at passage 3, at 60–70% confluency. It was centrifuged at 3500 rpm for 5 min to pellet down dead cells and cell debris. Then supernatants were filtered through 0.22 µm filters. The filtered supernatants were diluted with a SM (DMEM, 10% FBS, 1% antibiotics) in a ratio 1:1^26^. The SM for the experiments was prepared simultaneously in analogous manner. First portion was incubated for 24 h in clean culture dishes and then it was centrifuged, filtered, and diluted in a 1:1 ratio with fresh portion of medium.

### MTT assay

3-[4,5-dimethylthiazol-2-yl]-2,5-diphenyltetrazolium bromide (MTT) (Sigma-Aldrich, USA) was used to evaluate the viability of the LESC after exposure to LPS to determine the optimal concentration for LESC stimulation^27^. Absorbance referring to the concentration of formazan was measured using Wallac 1420 VICTOR plate reader (Perkin Elmer, USA) after 24 h of exposure to 0.1 µg/ml; 0.2 µg/ml; 0.5 µg/ml; 0.7 µg/ml; 1 µg/ml and 2 µg/ml of LPS and compared to control. All groups were analyzed in six replicates.

### Inflammation inducement

To mimic the inflammation, LESC were pretreated with LPS (lipopolysaccharide from *E. coli*, Sigma-Aldrich, USA) at concentration of 2 µg/ml for 24 h.

### ApoTox-Glo Triplex Assay

To measure cells viability, cytotoxicity and apoptosis in the samples, the ApoTox-Glo Triplex Assay (Promega, USA) was used according to the manufacturer’s instructions. Viability, cytotoxicity, and apoptosis of LESC were evaluated after 24 h exposure to examined conditions. LESC were firstly treated with CM and compared to SM to evaluate weather CM has beneficial impact to LESC activity. Then this evaluation was repeated after pretreatment with LPS (groups: SM_LPS, CM_LPS). Viability and cytotoxicity were measured by the fluorescent signal, which was emitted due to the cleavage of the added substrates glycyl-phenylalanyl-aminofluorocoumarin (GF-AFC) for viability and bis-alanyl-alanyl-phenylalanyl-rhodamine 110 (bis-AAF-R110) for cytotoxicity by specific proteases. GF-AFC enters the cells where it is cleaved via the live-cell protease activity to generate a fluorescent signal that is proportional to the number of living cells. AAF-R110 was used to measure the dead-cell protease activity, which was released from the cells that have lost membrane integrity. Both substrates have different excitation (400 nm, 485 nm) and emission (505 nm, 520 nm) spectra. Apoptosis was measured by the addition of a Caspase-Glo 3/7 Reagent. It is a luminogenic substrate that contains the tetrapeptide sequence DEVD in the reagent to assess caspase activity, luciferase activity and cell lysis. Fluorescence and luminescence were measured using a plate reader Triad LTMultimode Detector (Dynex Technologies, USA)^28^. All groups were analyzed in six replicates.

### Scratch assay

LESC’s proliferation rate was analyzed in all study groups (SM, SM_LPS, CM, CM_LPS). For this purpose, a scratch assay (wound healing test) was done. The analysis was performed bidirectionally. LESC were examined in standard condition and in induced inflammation at the same time. This assay had two control groups: SM for LESC cultured in standard condition and SM_LPS for LESC pretreated with LPS to induce the inflammation. At 80% of confluence LESC from group of induced inflammation were exposed to LPS for 24 h. LESC from standard condition group were at this time simultaneously cultured at incubator with no stimulation. After reaching the full confluence, the scratch was made using 200 µl pipette tip and cells were washed with PBS to remove non-adherent cells. After that medium was replaced for SM or CM^29^. Then the scratch area was photographed with an Olympus IX81 microscope (Japan) with a DP70 Olympus camera (Japan) and the wound area was measured using Image J software in time points: 0 h, 6 h, 12 h, 24 h, 36 h. All groups were analyzed in six replicates.

### Phenotype microarrays (PMM)

The phenotype of LESC was assessed under the influence of SM, CM and SM with the addition of LPS (SM + LPS) with phenotype microarrays (PMM, Biolog, USA) to evaluate whether CM or LPS have an impact on LESC phenotype and uptake of molecules to which it was exposed. Phenotype microarrays are 96-well microplates coated with different substrates in appropriate solvents e.g. ions, hormones and metabolic effectors. PMM show how cells metabolism, growth and productivity is affected by these agents. In order to the composition of substrates located on microarray, the PMM7 and PMM8 plates were used. These arrays are microplates coated with growth factors (e.g. IGF, FGF, PDGF), hormones (e.g. leptin, somatotropin, calcitonin, TSH) and cytokines (e.g. IL-1β, IL-6, IL-8, TNFα, IFNγ). This method enables the demonstration of cell preferences in the uptake of substrate added to the well of microarray. It also gives an information about possible cytotoxic effect of single compound. For this method cells previously were cultured in examined media for 24 h. Then, cells were collected by trypsinization and seeded on microarrays at the density of 10,000 cells per well. For the each medium (SM, CM, SM + LPS) one microarray of each type was used. LESC were cultured on microarrays for 24 h and after this period a dedicated tetrazolium salt (provided by the producer) was added to the wells for 3 h of further incubation. Added salt was reduced to soluble formazan what appeared in color-change reaction. The measured absorbance value corresponded to cell viability. The absorbance was measured with a Wallac 1420 VICTOR plate reader (Perkin Elmer, USA) at 590 nm^30^.

### Quantitative real-time polymerase chain reaction assay (real time RTqPCR)

Total RNA was extracted from LESC using a TRIzol reagent (Invitrogen, USA) according to the manufacturer’s instructions. The nucleic acid concentration was determined using a MaestroNano Spectrophotometer (Maestrogen, Taiwan). The expression genes related to inflammation (*IL6, IL-10, IL-2, IL-1α, IL-1β, IFNγ*) was detected using the real time RT-qPCR technique with SYBR Green chemistry (SensiFAST SYBR No-ROX Kit, Bioline, USA) and an Opticon DNA Engine Continuous Fluorescence detector (MJ Research Inc., USA) as was previously described^27^. For each biological replicate three technical replicates were performed.

### Enzyme-linked immunosorbent assay (ELISA)

The concentration of interleukins (IL-2, IL-4, IL-10, IL-6, IL-1α) secreted by LESC to the culture medium after exposure to the examined conditions, was analyzed with the use of immunoenzymatic tests (R&D Systems Quantikine ELISA Kits, USA) according to manufacturer protocol, as was described previously^31^. Optical density (OD) was read at 450 nm using Wallac 1420 VICTOR2 (PerkinElmer Inc., USA). For each biological replicate three technical replicates were performed.

### Statistical analysis

Statistical analysis was performed using Statistica 13.0 software (StatSoft, USA). A one-way ANOVA test with Tukey’s post hoc test and two-way ANOVA test or T-test were applied to evaluate any significant differences in the examined groups for normally distributed data. For non-normally distributed data Kruskal–Wallis test was applied. The level of significance was set at p < 0.05 for all statistical tests.

## Results

### Inflammation inducement

**Figure 1:**
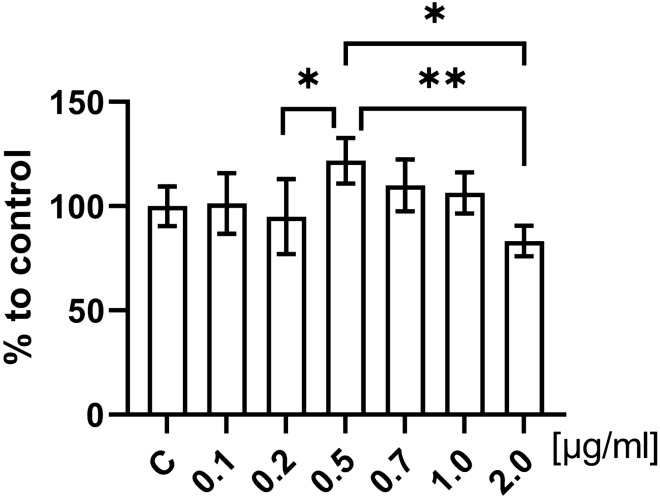
LESC viability based on the measurement of mitochondrial oxidative activity with MTT assay after exposure to LPS. The bars represent the means ± standard deviation (SD) of the percentages of the control group (fold to 100%); ANOVA with the Tukey post hoc test, *p < 0.05, **p < 0.01.

Viability of LESC was evaluated after 24 h of LPS treatment at following concentrations: 0.1 µg/ml; 0.2 µg/ml; 0.5 µg/ml; 0.7 µg/ml; 1 µg/ml and 2 µg/ml in comparison to control (0 µg/ml of LPS). Control cells were taken as 100% (Fig. 1.). We did not observe a statistically significant differences in the mitochondrial activity in the tested cells, which confirms that LPS is not toxic to LESC in the studied range of its concentrations. However, meaningfully decreased viability was observed in 0.2 µg/ml (p = 0.0337) and 2 µg/ml (p = 0.0009) groups compared to cells treated by 0.5 µg/ml of LPS. In addition, viability of 2 µg/ml group was also lower in comparison to 0.7 µg/ml group (p = 0.0351). Based on these observations, the LPS concentration of 2 µg/ml was selected as optimal and nontoxic for the inflammation inducement in LESC for further analyzes.

### Effect of ADSC secretome on viability, cytotoxicity, and apoptosis of LESC

**Figure 2:**
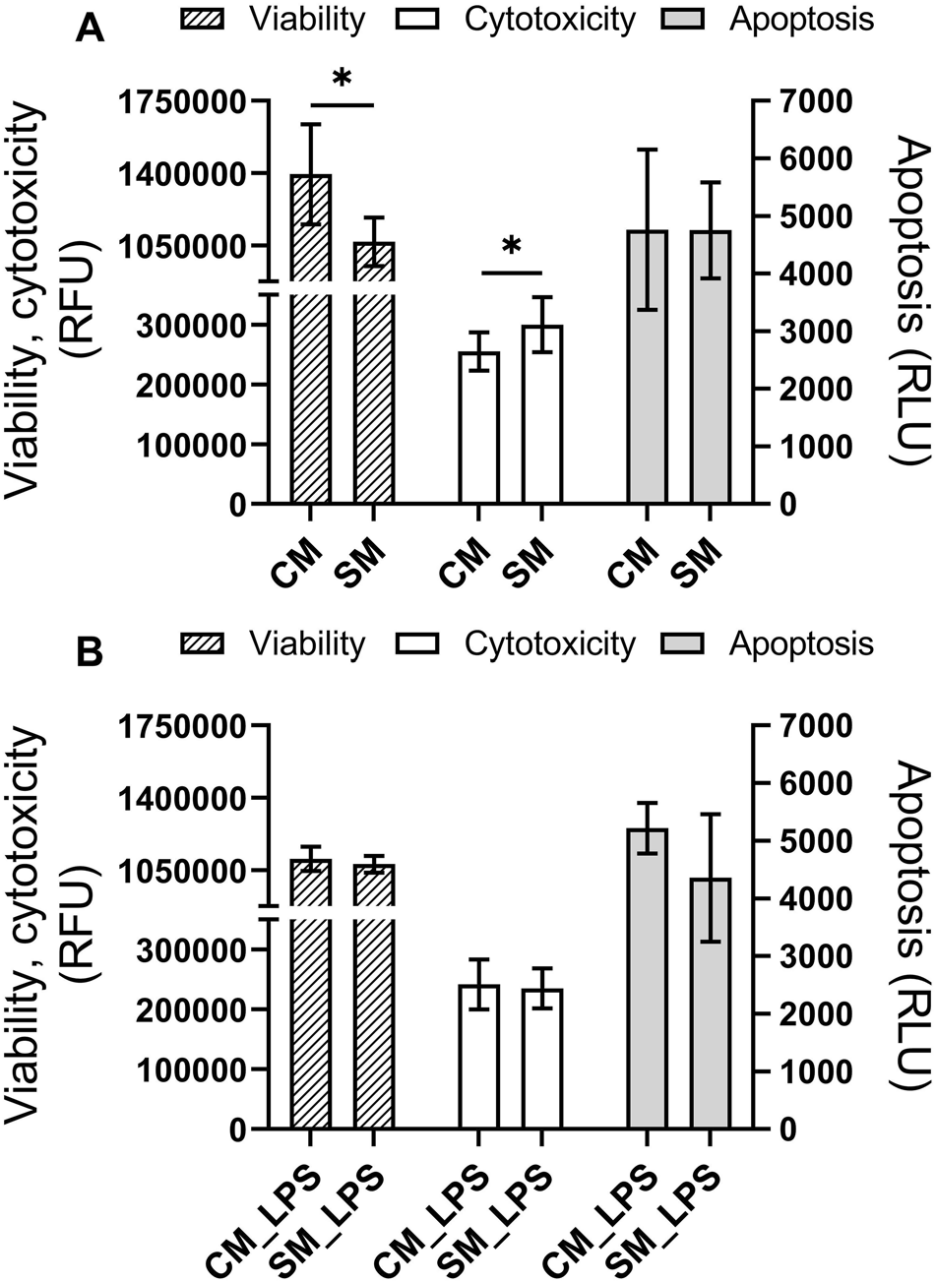
Viability, cytotoxicity and apoptosis of LESC in response to conditioned medium from ADSC (CM) in standard condition and in induced inflammation (LPS groups). The activity of the dead-cell proteases (viability and cytotoxicity) and the activity of caspase 3/7 (apoptosis) are shown as relative fluorescence units or the relative luminescence units. (**A**) CM compared to SM group. (**B**) CM_LPS compared to SM_LPS group. *SM* standard medium. The bars represent the means ± standard deviation (SD). T-test, *p < 0.05.

LESC treated with CM showed a significant increase in viability (p = 0.023) and decrease in cytotoxicity (p = 0.033) comparing to the control group (SM). We have not observed significant changes in the apoptosis of LESC cultured in SM and CM. This suggest that CM improves LESC living. We did not find statistically significant differences in viability, cytotoxicity and apoptosis of cells pretreated with LPS (Fig. 2), what suggest that LPS disturbs LESC’ response to the beneficial activity of CM.

### Impact of ADSC secretome on proliferation and migration of LESC

**Figure 3:**
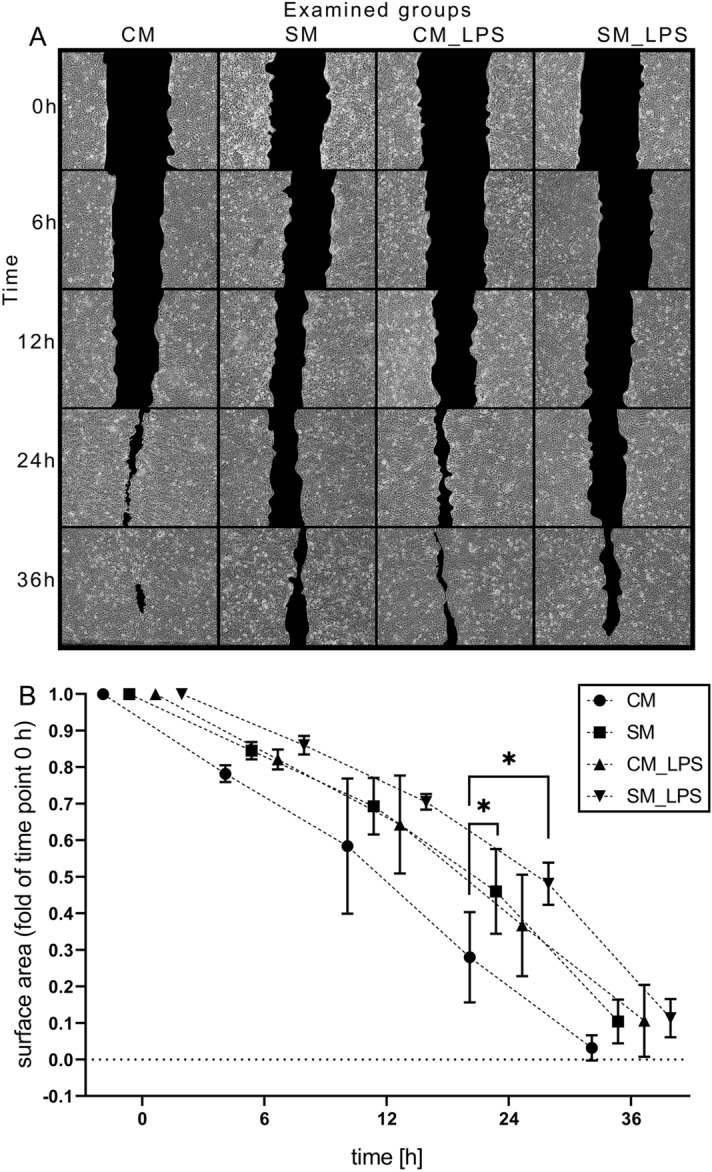
(**A**) Scratch assay of LESC monolayer in response to conditioned medium from ADSC (CM) in standard condition and in induced inflammation (LPS groups). The results present a one selected microscopic image from six replications of each group (CM, SM, CM_LPS, SM_LPS) at time points (0 h, 6 h, 12 h, 24 h, 36 h). Scratch area marked with black. (**B**) Scratch surface area as fold of time point 0 h of examined groups (CM, SM, CM_LPS, SM_LPS) in time (6 h, 12 h, 24 h, 36 h). Two-way ANOVA test, *p < 0.05.

Scratch assay was made to assess an influence of ADSC secretome on LESC in standard condition and induced inflammation. The results of microscopic observations are presented in the Fig. 3A. Wound areas were calculated as the fold change of measured surface area (total number of pixels, N = 6) in examined groups (CM, SM, CM_LPS, SM_LPS) after 6 h, 12 h, 24 h and 36 h compared to the initial time point (0 h). The two-way analysis of variance (ANOVA, p < 0.05) revealed significant differences between the examined groups and showed that cells treated with CM had higher proliferation rate compared to the cells grown in SM, both in standard condition and in induced inflammation. However, the difference between CM_LPS and SM_LPS was not statistically significant. It was noticed that cells treated with LPS showed lower proliferation rate than non-stimulated cells, (Fig. 3B).

### Impact of ADSC secretome on phenotype of LESC

**Figure 4:**
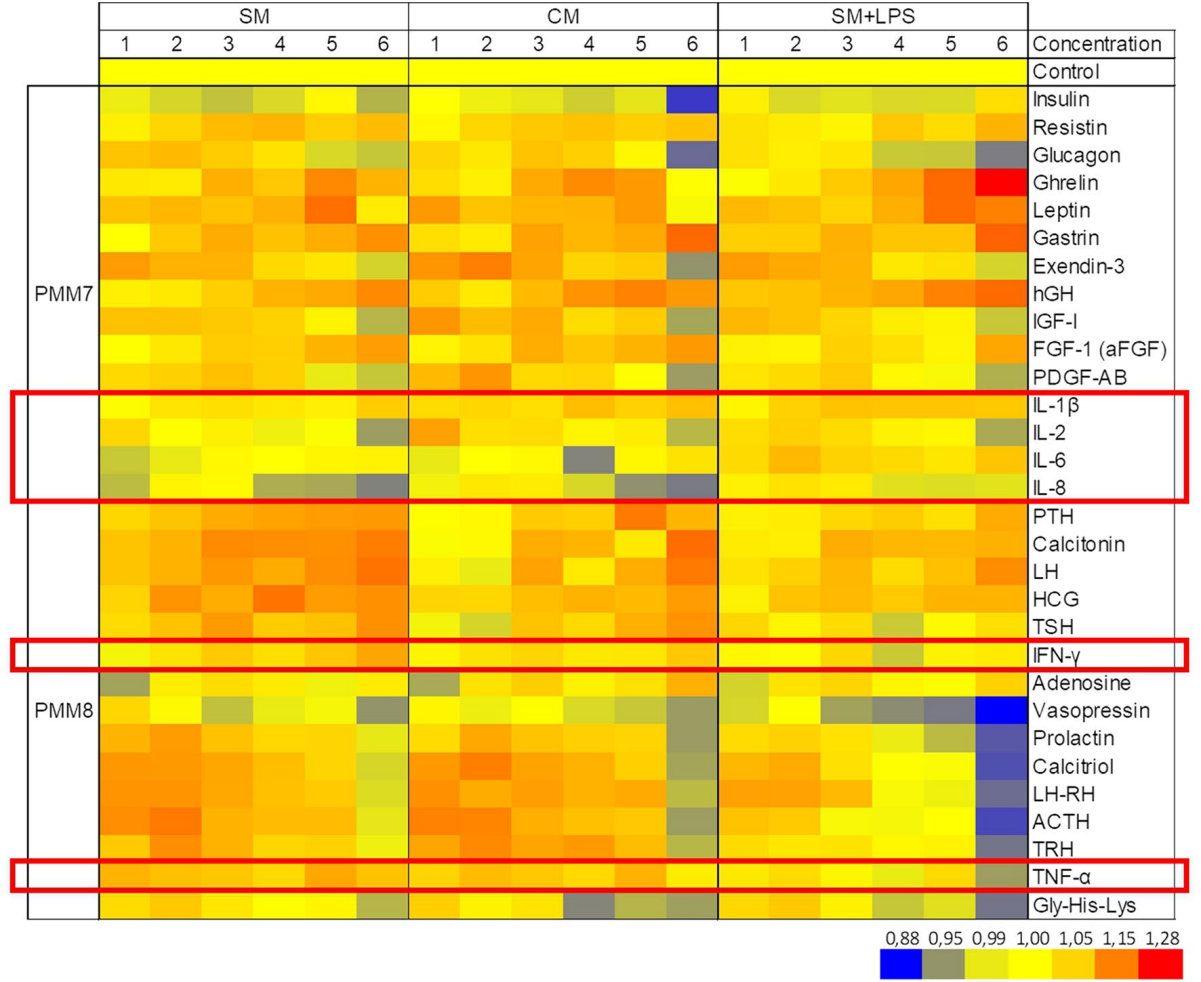
Heatmap of fold change in Phenotype MICROARRAYS for mammalian cells, PMM7 and PMM8—“MICROPLATE—Hormones & Metabolic Effectors” in examined groups. *SM* standard medium, C*M* conditioned medium from ADSC, *SM + LPS* standard medium with the addition of 2 µg/ml of LPS. Red—highest values, blue—lowest values, yellow—neutral. Cytokines are marked with red frame. This heatmap was created with MS Excel 2019 software.

Obtained results are presented in heatmaps (Fig. 4). The pattern of heatmap for each group significantly differs what shows that the preferences of cells have changed due to the impact of CM or LPS. A detailed analysis showed a diverse reaction to the presence of hormones and metabolic effectors (growth factors and cytokines) present on microarrays, such as: insulin, resistin, glucagon, ghrelin, leptin, gastrin, exendin-3, hGH, IGF-I, FGF-1, PDGF-AB, IL-1β, IL-2, Il-6, Il- 8, PTH, calcitonin, LH, HCG, TSH, IFN-γ, adenosine, vasopressin, prolactin, calcitriol, LH-RH, ACTH, TRH, TNF-α, and Gly-His-Lys tripeptide. LESC showed decreased activity in the presence of insulin, IL-8, and prolactin in all examined groups. Cells cultured in CM showed less activity in response to IL-6 and the Gly-His-Lys tripeptide. Whereas cells after the culture in SM showed decreased activity in response to IL-2. Finally, cells cultured in SM + LPS showed less activity in the presence of glucagon. The above results varied in lower concentrations. A detailed description of our findings is included in discussion.

### Impact of ADSC secretome on the transcriptome of LESC

**Figure 5:**
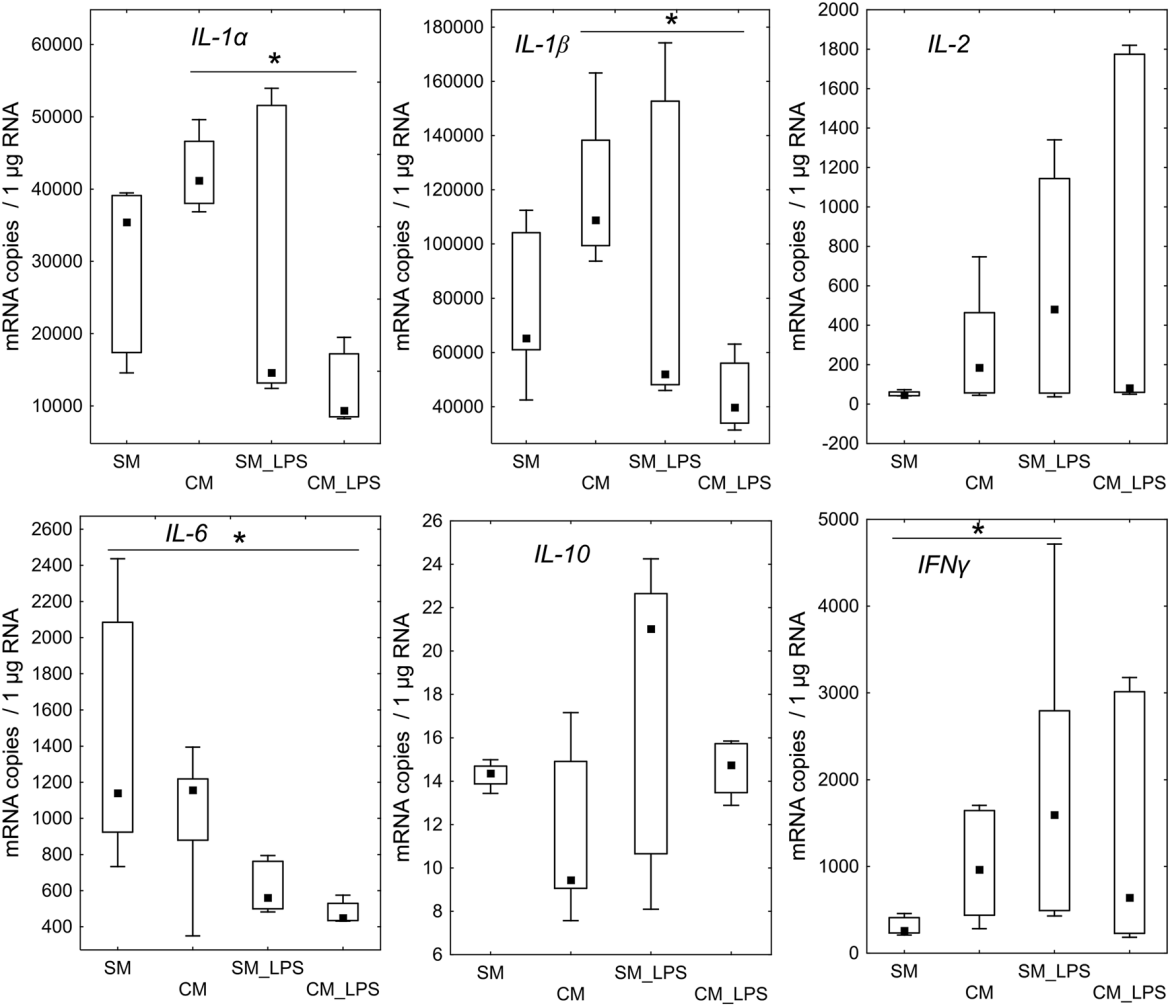
The mRNA levels of inflammation associated genes coding: *IL-1α, IL-1β*, *IL-2*, *IL-6*, *IL-10*, *IFNγ*. The bars represent the (Me) with the 25th and 75th quartiles and the minimum and maximum of the copy numbers per 1 µg of total RNA. The Kruskal Wallis test with post hoc analysis was applied to assess any differences between groups in the expression of the genes, *p < 0.05.

The expression of genes in LESC coding pro-inflammatory cytokines *IL-1α* (p = 0.0075) and *IL-1β* (p = 0.0098) was significantly lower in LESC cultured in CM after pretreatment with LPS (CM_LPS) in comparison to LESC cultured in CM. The *IL-6* mRNA level was lower in CM_LPS group (p = 0.0177) versus SM. Level of *IFNγ* mRNA in SM_LPS was higher (p = 0.0374) than in SM group. *IL-2* and *IL-10* mRNA did not differ in analyzed groups (Fig. 5.).

### Impact of ADSC secretome on inflammatory cytokines secretion by LESC

**Figure 6:**
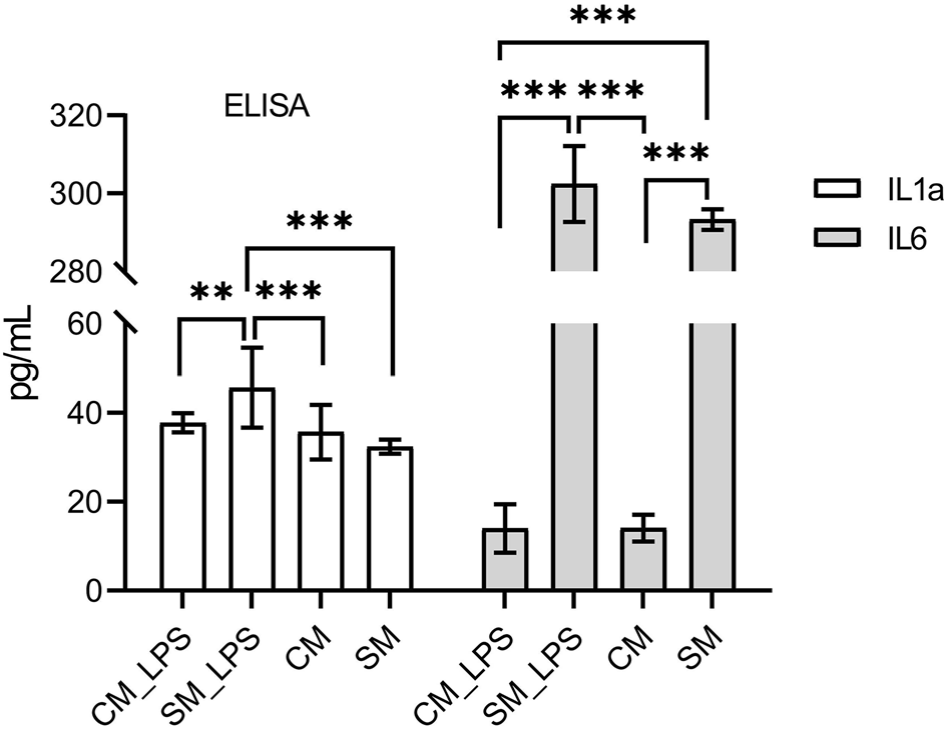
Secretion of cytokines by LESC to the culture medium in all examined groups (CM, SM, CM_LPS, SM_LPS) measured by ELISA. ANOVA with the Tukey post hoc test, **p < 0.01, ***p < 0.001.

The secretion of cytokines (IL-2, IL-10, IL-6, IL-1α, IFNγ and IL-4) to the culture medium by LESC was quantified by ELISA. Only the presence of IL-6 and IL-1α was detectable in cell medium. The secretion of IL2, IL10, IL4 and INFy has not been noticed. (Fig. 6). The concentration of IL-6 was significantly lower in LESC cultured in CM both in standard condition (p = 0.0002) and in induced inflammation (CM_LPS) (p = 0.0002) compared to SM and SM_LPS groups. The concentration of IL-1α was significantly lower both in CM (p = 0.0006) and CM_LPS (p = 0.0066) groups compared to SM_LPS. We also noticed lower level of IL-1α in SM (p = 0.0002) group compared to SM_LPS. There were no differences between CM, CM_LPS and SM groups.

## Discussion

The paracrine activity of ADSC has been investigated for several years. Its beneficial impact on surrounding cells has been reported as potentially useful for random applications associated with e.g. tissue regeneration, immunomodulation, skin graft survival improvement or eye diseases treatment^32–36^. ADSC can be easily isolated from patients’ body due to the abundant presence of fat tissue from where they can be gathered. Although these cells are potentially available at any time, there are limitations in their use. ADSC show in vitro high proliferation rate at early passages which decreases directly proportionally to the time of cells culture and further cell passages^37^. It is recommended to keep primary ADSC for the short period in culture dishes and use them at low passages, however the period of healthy ADSC culture can be extended by supplementing the culture medium with antioxidants^38,39^. It has been reported that lowering calcium concentration and culturing ADSC under hypoxia also affects their proliferation and differentiation, what should be considered when planning the long-term cell culture^40–43^. Our previous reports indicated that the environment in which ADSC are maintained strongly affects their phenotype and in vitro activity such proliferation or differentiation^27,44,45^. It is known that the environment has also a strong impact on paracrine activity of ADSC^46^ what depends on the profile of secreted molecules among which are i.e. cytokines, growth factors^47^, exosomes containing microRNAs or organelles like mitochondria^48,49^. It was reported that the paracrine activity of mesenchymal stem cells (MSC) such ADSC is regulated by internal signaling pathways with meaningful role of the Rap1 (Trf2IP—a telomeric repeat-binding factor 2-interacting protein 1) which modulates the activity of NF-κB^50–52^.

Fat tissue is not the only source of MSC. MSC are being isolated from bone marrow, Wharton’s jelly, amniotic membrane, dental pulp, amniotic fluid and more^53,54^. Various sources of MSC, patients’ health or cell culture conditions are one of the factors which have strong impact on MSC’ secretion profile what leads to the differences between the following batches of collected CM for potential therapy. Moreover, source-dependent limitations using MSC is being tried to overcome by de-differentiation of somatic cells, also MSC, into the iPSC (induced pluripotent stem cells). IPSC can be multiplied unlimitedly and then differentiated into the MSC again^55–58^. This practice could ensure the consistency of conditioned medium quality.

Above concerns should been considered before starting a cell culture for the collection of CM from ADSC at best possible conditions. Priming cells with optimized culture medium or introduction of genetic engineering methods for regulating Rap1/NF-κB pathway activity could benefit with better outcome of ADSC paracrine activity by induced secretion profile. The potential use of CM from ADSC require strict procedure of its collection and standardization which should ensure the stable paracrine profile of ADSC and consistent quality of CM.

In this study our objective was to assess the direct influence of CM from ADSC on limbal epithelial stem cells in several stages. The first stage included the choice of lipopolysaccharide concentration for the inducement of inflammation. The effect of a series of concentrations of LPS on LESC was assessed, however MTT assay showed no statistically significant differences between the examined groups compared to the control. Similarly Kukolj et al.^59^ observed that LPS did not affect proliferation and viability of periodontal ligament stem cells and it did not change their immunophenotype and cell cycle. Kukolj et al. showed that LPS acts on differentiation potential of cells and it inhibits osteogenesis and promotes chondrogenesis and adipogenesis^59^. Cell viability may change under the influence of LPS depending on the time of incubation and its concentration^60^. Many studies suggest the application of different concentrations from very low like 0.1 μg/ml^61^; through 0.5 μg/ml^62^, 1 μg/ml^60^ to 10 μg/ml^60,61^ or even 24 μg/ml^63^. Moreover, the suggested incubation time which is necessary for the induction of inflammation is diverse and takes e.g. from 2 to 24 h^60^. Some studies indicate that a proper time to induce the inflammation in mesenchymal stem cells is 24 h with 1 μg/ml of LPS concentration^64^. Other suggest increasing the concentration of LPS to 2 μg/ml, but shortening the incubation time to 6 h in morphologically similar cells like normal human skin fibroblasts (NHDF)^65^. In contrast, LPS at concentration of 0.1 μg/ml was used for the induction of inflammation in macrophages for 24 h^66^. Therefore, based on numerous literature data and our findings, the concentration selected for this experiment was 2 µg/ml of LPS.

Next, the analysis with ApoTox-Glo Triplex Assay showed that LESC viability increased after the treatment of conditioned medium from ADSC. The test showed also that the CM was not toxic to cells and as well there were no differences in cells apoptosis between groups. These results suggest that factors secreted by ADSC improve LESC’ viability. In the condition of induced inflammation, no statistically significant differences in cell viability, cytotoxicity and apoptosis were noticed under the influence of CM. These findings suggest that LPS inhibits the positive effect of ADSC’ secretome on limbal stem cells. Probably different signaling pathways were activated in the presence of LPS in the cells, not necessarily associated with the promotion of cell viability. It indicates that the addition of LPS does not significantly reduce cell activity. Probably a longer cell culture in the above-mentioned conditions could result in similar findings as in the case of cells cultured in standard condition. However, these results show that the presence of LPS changes the activity of cellular proteases in LECS. Similar findings were reported by Chen and his team who investigated the effect of conditioned medium from mesenchymal stem cells derived from Wharton jelly on epithelial cells. It was shown that conditioned medium increased cells proliferation and migration by activation of stress response kinase JNK (c-Jun N-terminal Kinase) and isoform of p38 protein^67^. Farahmand et al. also confirmed these results by observing higher expression of proliferation-related genes after culture in conditioned medium^68^. Some data indicated using a conditioned medium in the therapy of e.g. arthritis. In this case, a reduction of damages in cartilage tissue was shown and an inhibition of the immune response in cells has been noted^33^. Li et al. investigated using conditioned medium as a promoter of wound healing in diabetic patients in whom this process is usually disturbed. Research showed that the use of LPS reduces the rate of migration and proliferation in keratinocytes which grown in the presence of high glucose concentration, as the disease model for analyzing the effect of type 2 diabetes on cells. It was proven that the use of CM eliminates the negative effect of LPS and also negative effect of high glucose levels^69^. Also, the high potential of using CM in the treatment of bronchopulmonary dysplasia in oxygen-induced alveolar damage model showed that MSCs support tissue with antioxidant substances which therefore can lead to new therapy^70^.

Scratch wound healing assay was performed to assess the rate of cell proliferation. LESC cultured in CM compared to SM, both under standard conditions and induced inflammation, showed better proliferation rate. It was noticed that cells stimulated with LPS and cultured in CM showed lower proliferative potential compared to unstimulated cells. These results indicate inhibition of cells proliferation by LPS, which is consistent with Apo-Tox-Glo assay’s indications and the literature data^69^. Zhang et al. in in vivo studies showed that injection of ADSC promoted wound healing in rabbits^71^. Zeppieri et al. presented that mesenchymal stem cells promote corneal wound healing in rats^72^. Similarly, Galindo et al. demonstrated that ADSC have a therapeutic effect on LSCD in rabbits^73^.

Phenotype microarrays showed that LESC in all study groups (SM, CM, SM + LPS) had various activity. PMM7 and PMM8 arrays were coated with six replicates of the same compound but in variable concentration. Producer provides an information that these concentrations are increasing from left to right but does not give the information about the number and the unit, so we do not really know how much of each compound is in the well. We have numbered these concentrations from 1 to 6, where 1 is the lowest concentration and 6 is the highest. Due to a lot of data on cells activity, the results are presented on heat maps what ensures clear readability. All readings are presented as the fold change compared to control which was taken as 100%. We noticed that the activity of LPS significantly impact on LESC viability. This group of cells became more sensitive for bigger concentrations of each compound what appeared in the highest spread of fold change values. Interestingly, we noticed that LESC cultured in SM with the addition of LPS were more viable in presence of IL-6, while LESC cultured in SM and CM showed neutral or toxic effect of this cytokine. The lowest viability in presence of IL-6 was noticed in CM. We assume that while ADSC secrete IL-6, the final concentration of this cytokine in one well could be high enough to act negatively on LESC^32,74,75^. Surprisingly, the highest toxic effect was observed at the concentration “4”. LESC showed decreased activity in response to IL-8 in all examined groups. The toxic effect was more readable in higher concentrations and the highest values were noticed in CM group, but there were no differences between SM. Similarly, IL-2 in higher concentrations showed a toxic effect on LESC viability in all groups, but in opposite, lower concentrations of IL-2 induced LESC activity what was mostly notable in CM group. We observed that INFγ exerted a neutral response of LESC. In SM and CM groups we noted slightly increasing viability in direct proportion to the INFγ concentration. LESC cultured in SM + LPS showed decreased viability in the presence of INFγ.

Phenotype microarrays brought plenty of information which could help composing supplementation of culture media to improve cells viability. These factors could be potentially cross-linked in biopolymer scaffolds adapted for carrying ADSC to strengthen the response to secreted factors. However, further studies on the influence of presented factors are needed for better understanding of variable effect on LESC activity.

The last stage included the assessment of mRNA expression and cytokine secretion of pro and anti-inflammatory cytokines in LESC due to the examined conditions. Increased expression of *IL-1α* and *IL-1β* was observed in cells cultured in CM at standard conditions compared to cells with induced inflammation (CM_LPS). IL-1α and IL-1β role is associated with the regulation of an immune response as a result of ongoing infection^76,77^. IL-1 also acts by activating the secretion of many different cytokines and chemokines, e.g. IL-6, TNFα and IFNγ^76,77^. IL-1 participate in the regulation of stem cell activity. In hematopoietic stem cells (HSCs), it exhibits radioprotective activity, induces their proliferation and differentiation^77^. Our findings showed no differences in the expression of *IL-1α* and *IL-1β* between cells cultured in CM and SM in both standard condition and induced inflammation. The lower expression of both interleukins in CM_LPS group suggests that CM has a significant impact on IL-1 release during the inflammation. This activity was confirmed by ELISA detection of IL-1α. The concentration of this IL-1α in LESC’ culture medium was lower during induced inflammation under the influence of CM (CM_LPS group). Furthermore, significant difference in the secretion of IL-1α between LECS with induced inflammation and LESC in standard condition cultured in SM indicates that LPS has meaningful impact on IL-1α release. Finally, observed no differences in IL-1α secretion between CM, CM_LPS and SM group suggest that CM downregulates the release of IL-1α which means that ADSC secrete factors which mitigates the inflammation. Some studies suggest that an increase in interleukin-1 secretion was observed under the influence of cellular stress e.g. in a state of hypoxia, chemical or physical damage. During the apoptosis which is natural, programmed cell death IL-1α is not released by the cells^76^. Solomon et al. reported that stroma of amniotic membrane inhibits the level of IL-1α and IL-1β, after LPS stimulation, in cell culture of limbal epithelial stem cells^78^. It suggests that the use of CM simultaneously with amniotic membrane could give better therapeutic results in cornea treatment by achieving a synergistic effect. The analysis of the *IL-6* mRNA expression showed decreased copy number in cells with induced inflammation cultured in CM compared to SM. ELISA also indicated a significant decrease of IL-6 secretion by LESC with induced inflammation compared to cells cultured in SM. Similarly, lower concentration of IL-6 in the same group was noticed at LESC in standard conditions. Studies indicate that IL-6 secretion increase in cells during inflammation^79,80^. However, it was proven that MSC naturally secrete IL-6^81,82^. This interleukin is responsible for the regulation of the immune response, hematopoiesis, apoptosis, proliferation and cell viability^83^. It plays important role in regulation of homeostasis in corneal limbus cells niche. IL-6 may help in wound healing of epithelial cells in vivo^84,85^. Perhaps a significant increase in the level of this interleukin may be the result of cellular responses to unfavorable environmental conditions e.g. like LPS induced inflammation. Lower levels of IL-6 at LESC cultured in CM suggest a beneficial property of ADSC secreted factors. Probably factors present in the conditioned medium from ADSC promote the LESC regeneration. Perhaps the use of IL-6 as a supplement for cell culture could provide better proliferation. However, this issue requires further study. The analysis also revealed an increase of *IFNγ* mRNA expression in LESC with induced inflammation cultured in SM compared to the LECS in standard condition cultured in the same medium. This indicates that LPS stimulation probably caused upregulation of this gene expression. However, IFNγ was not detected by ELISA. It should be remembered that the increase in mRNA level is ahead of the protein concentration. Gene expression depends on many transcription factors which activation changes the level of mRNA in cells. Thus, when translation product is at demanded level, the DNA transcription can be suppressed. In opposite, when there is a low level of protein, gene expression can be continuously upregulated.

## Conclusion

Our results showed that the paracrine activity of ADSC on LESC promotes its proliferation. There is visible impact of ADSC on LESC viability and proliferation in the condition of inflammation induced by LPS. However, this role of ADSC activity on LESC require further investigation and the analyzes with bigger study groups. LPS has a meaningful impact on LESC metabolism. We find the ADSC’ secreted factors useful for LESC regeneration. Placing the ADSC in closed carrier can probably be used for temporal wound dressing. The ADSC’s secretome probably could be used in development of new medicinal products for corneal injures.
